# Molecular Characteristics of Bovine Viral Diarrhea Virus Strains Isolated from Persistently Infected Cattle

**DOI:** 10.3390/vetsci10070413

**Published:** 2023-06-25

**Authors:** Yinghao Wu, Guangzhi Zhang, Hui Jiang, Ting Xin, Li Jia, Yichen Zhang, Yifei Yang, Tong Qin, Chuang Xu, Jie Cao, Gobena Ameni, Arfan Ahmad, Jiabo Ding, Limin Li, Yuzhong Ma, Xuezheng Fan

**Affiliations:** 1 College of Veterinary Medicine, Hebei Agricultural University, Baoding 071001, China; yinghaowu1618@outlook.com (Y.W.); jiali18033630734@outlook.com (L.J.); lilimin03@hebau.edu.cn (L.L.); 2 Institute of Animal Sciences, Chinese Academy of Agricultural Sciences, Beijing 100193, China; zhangguangzhi@caas.cn (G.Z.); jianghui01@caas.cn (H.J.); xinting@caas.cn (T.X.); 82101215579@caas.cn (Y.Z.); qintong@caas.cn (T.Q.); dingjiabo@caas.cn (J.D.); 3 College of Veterinary Medicine, China Agricultural University, Beijing 100193, China; baiyi@cau.edu.cn (Y.Y.); xuchuang@cau.edu.cn (C.X.); caojie@cau.edu.cn (J.C.); 4 Department of Veterinary Medicine, College of Agriculture and Veterinary Medicine, United Arab Emirates University, Abu Dhabi P.O. Box 15551, United Arab Emirates; gobena.ameni@uaeu.ac.ae; 5 University Diagnostic Lab, University of Veterinary & Animal Sciences, Lahore 54000, Pakistan; iffivet@uvas.edu.pk

**Keywords:** bovine viral diarrhea virus, persistently infected cattle, BVDV-1b, molecular characteristics

## Abstract

**Simple Summary:**

Bovine viral diarrhea virus (BVDV) is a pathogen that is widespread throughout the world, causing serious economic losses. Most of the current BVDV infection research focuses on genotyping based on the 5′-UTR sequence, neglecting the molecular characteristics of the whole genome of BVDV strains. Cows infected with non-cytopathic (NCP) BVDV in early pregnancy may give birth to persistently infected (PI) cattle, which are the main sources of virus transmission in the herd. The present study isolated and identified nine BVDV strains from PI cattle in China. The complete genome of the new isolates was sequenced for phylogenetic analysis, recombination analysis and sequence analysis in 5′-UTR, E0 and E2.

**Abstract:**

In this study, we reported the isolation, identification, and molecular characteristics of nine BVDV strains that were isolated from the serum of persistently infected cattle. The new strains were designated as BVDV TJ2101, TJ2102, TJ2103, TJ2104, TJ2105, TJ2106, TJ2107, TJ2108 and TJ2109. The TJ2102 and TJ2104 strains were found to be cytopathic BVDV, and the other strains were non-cytopathic BVDV. An alignment and phylogenetic analysis showed that the new isolates share 92.2–96.3% homology with the CP7 strain and, thus, were classified as the BVDV-1b subgenotype. A recombination analysis of the genome sequences showed that the new strains could be recombined by the major parent BVDV-1a NADL strain and the minor parent BVDV-1m SD-15 strain. Some genome variations or unique amino acid mutations were found in 5′-UTR, E0 and E2 of these new isolates. In addition, a potential linear B cell epitopes prediction showed that the potential linear B cell epitope at positions 56–61 is highly variable in BVDV-1b. In conclusion, the present study has identified nine strains of BVDV from persistently infected cattle in China. Further studies on the virulence and pathogenesis of these new strains are recommended.

## 1. Introduction

Bovine viral diarrhea virus (BVDV), the pathogen that causes bovine viral diarrhea mucosal disease (BVD-MD), is a member of the Pestivirus genus in the Flavivirus family [1]. Classical swine fever virus (CSFV) and border disease virus (BDV) are members of the same family [2]. In addition to bovines, BVDV can also infect 50 species, such as sheep, swine, giraffes, and camels, inducing viral diarrhea characterized with pyrexia, diarrhea, leukopenia, abortion, and immunosuppression [3]. The 5′ untranslated regions (5′-UTR) among the same genotype are highly conserved. Based on the comparison of 5′-UTR sequences, BVDV can be grouped into three genotypes and 30 subgenotypes (BVDV-1a~1v, BVDV-2a~2d, BVDV-3a~3d) [4,5]. Depending on the presence or absence of the cytopathic effect (CPE) in BVDV-infected cells, BVDV can also be divided into two biotypes; the cytopathic (CP) biotype and non-cytopathic (NCP) biotype [6,7].

BVDV is a positive sense single stranded RNA virus with a genome of about 12.3 kb. The genome contains a single open reading frame (ORF) flanked by the 5′ and 3′ untranslated region (UTR) [8,9]. 5′-UTR is a highly conserved sequence consisting of 360–386 nucleotides, and it has been used to identify virus subgenotypes together with N^pro^ and the E2 region [4,10]. The ORF encodes a polyprotein composed of 3900 amino acids. The polyprotein is sequentially encoded by different regions of the ORF and then processed by cellular and viral proteases into four structural proteins (C, E0, E1, and E2) and seven or eight non-structural proteins (N^pro^, P7, NS2-3 or NS2, NS3, NS4A, NS4B, NS5A, and NS5B) [11,12]. NS2 is a cysteine autoprotease that can catalyze the cleavage of NS2-3 into NS2 and NS3. In the early stage of infection, the cleavage of NS2-3 can be conducted effectively in both NCP and CP BVDV infected cells. However, at 6–9 hpi, NS2-3 auto-processing decreases to an undetected level for NCP BVDV, while only decreasing moderately for CP BVDV [13,14]. In addition to NCP BVDV, CP BVDV can also be isolated from persistently infected animals. It has been confirmed that CP BVDV strains are often derived from NCP BVDV strains in PI cattle through genomic mutations. These changes could be insertions of ubiquitin mRNA coding sequence (NEDD8, LC3) or the J-domain protein, which could maintain the NS2-3 auto-processing and contribute to cytopathic mutation [15,16,17,18].

Glycosylation is one of the modification methods of complex proteins, which affects the stability and antigenicity of protein and protein–protein interactions [19]. E0, E1 and E2, three glycoproteins of BVDV, play important roles in the process of cell recognition, cell invasion and the formation of virus particles [20,21]. E0 protein is a glycosylated protein composed of 227 amino acids with a mass of about 44–48 kD [22]. As an envelope protein, E0 is relatively conserved in BVDV genome proteins and has good antigenicity, so it can be used as an alternative antigen for subunit vaccine preparation. In addition, BVDV E0 interacts with glycosaminoglycans (GAGs) and attaches to cells, which is the first step during virus infection [23,24]. E2 protein is a type I transmembrane protein composed of about 370 amino acid residues and has a mass of 55 kD [25]. Glycosylation sites of viral membrane proteins affects viral pathogenicity and antigenicity. During the process of virus assembly, E2 combines with E1, or itself, into an E1-E2 or E2-E2 dimer, which participates in the formation and fusion of virus particles. With the strongest antigenicity in BVDV proteins, E2 can induce the host’s B lymphocytes to produce neutralizing antibodies, which is the focus of BVDV vaccine research [26,27,28].

In recent years, the increased domestic demand for beef and dairy products has accelerated trading and the development of the cattle industry. Since the Changchun 184 strain was originally isolated from an aborted bovine fetus in 1980, BVDV infection has spread widely in China, almost all over the country. The ten subgenotypes found in China include 1a, 1b, 1c, 1d, 1m, 1o, 1p, 1q, 1u, and 1v; of which 1a, 1c and 1m were dominantly found to be contributing to 31.1%, 28.6% and 21.0% of the total subgenotypes, respectively [5]. In addition, the total positive rate of the BVDV antibody in China, in clinical healthy dairy cattle, beef cattle, buffalo and yak, was 58.09% [29]. These data indicate that BVDV infection has a high prevalence throughout China with high genetic diversity. The pregnant cows infected with NCP BVDV during 30d–150d of gestation may give birth to persistently infected (PI) calves, which are immunotolerant against BVDV; therefore, clinical tests based on immune responses become ineffective [30,31,32]. PI cattle carry the virus for life and are responsible for the spread of BVDV throughout herds via continuous viral shedding from excrement and secretions [33]. In this study, we successfully isolated nine BVDV strains from the serum of calves with diarrhea. To understand the genetic characteristics and possible evolutionary origins of circulating BVDV strains, we amplified and analyzed their whole genome sequences, and reported the molecular characteristics of the new isolates. These findings could contribute to the prevention and control of BVDV.

## 2. Materials and Methods

### 2.1. Cell Culture and Virus Isolation

Madin–Darby Bovine Kidney (MDBK) cells were grown in DMEM (Gibco, New York, NY, USA) containing 10% BVDV-free fetal bovine serum (Royacel, Lanzhou, China). The serum samples were obtained from PI cattle with mucosal disease at a field in Tianjin in 2021. The PI cattle were tested to be positive for the BVDV antigen and negative for antibodies. For virus isolation, the serum samples were diluted one-fold with 10 mM phosphate buffered saline (PBS), then were filtered with a 0.22 μm filter before virus infection. Monolayer MDBK cells were infected with the treated samples in 6-well culture plates. The cytopathic conditions were observed and recorded every day. After 96 h, the infected cells were lysed using three cycles of freeze-thaw, and centrifuged at 5000 rpm for 3 min. Then, the supernatant was taken for the next infection. The 5th passages of viruses were used for further research.

### 2.2. Indirect Immunofluorescence Assay (IFA)

To identify the isolated BVDV strains, monolayer MDBK cells were infected with the 5th passages of the isolated viruses. Mock-infected cells were used as the negative controls. After culturing for 48 h, the cells were fixed with 4% paraformaldehyde, incubated with 0.2% TritonX-100, and blocked with 1% bovine serum albumin. Then, the cells were incubated with BVDV monoclonal antibodies (VMRD, Washington, WA, USA) and Goat anti-Rabbit IgG/FITC (Solarbio^®^, Beijing, China) before examination using the fluorescence microscope.

### 2.3. RNA Extraction and RT-PCR

Total RNA was extracted from the lysate of infected cells using a TransZol Up Plus RNA Kit (TransGen, Beijing, China) according to the manufacturer’s instructions. Briefly, the samples were lysed using a triple volume of TransZol reagent, mixed with 0.2 volume of an RNA Extraction Agent, and shaken vigorously for 5 min. After centrifugation at 10,000× *g*, 4 °C for 15 min, the upper aqueous phase was washed with anhydrous ethanol. Then, the RNA was resuspended in 30 μL Diethyl pyrocarbonate (DEPC) treated water. Reverse transcriptase reactions were carried out using EasyScript One-Step gDNA Removal and cDNA Synthesis SuperMix (TransGen, Beijing, China) in a 20 μL reaction volume containing 6 μL of extracted RNA, 1 μL Anchored Oliogo(dt)18 Primer, 10 μL of 2 × ES Reaction Mix, 1 μL of EasyScript RT/R1 Enzyme Mix, 1 μL of gDNA Remover, and 1 μL of RNase-free Water. The following reaction was 42 °C for 30 min and 85 °C for 5 s. PCR amplification was conducted using the 2 × Pfu PCR Mix (Tiangen, Beijing, China) in a 20 μL reaction volume containing 10 μL of 2 × Pfu PCR Mix, 1 μL of upstream and downstream primers (5′-TCAGCGAAGGCCGAAAAGAGG-3′, 5′-TCCATGTGCCATGTACAGCAGAG-3′), 2 μL of cDNA template, and 6 μL ddH_2_O. The following reaction was 94 °C for 5 min, followed by 35 cycles at 94 °C for 30 s, 46 °C for 30 s, and 72 °C for 1 min, and an extension at 72 °C for 10 min.

### 2.4. Complete Genomic Amplification and Sequencing

Twelve pairs of primers (Table A1) were designed using the Primer Premier 5 according to the BVDV JL-1 strain (GenBank No. KF501393.1) and BVDV CC13B strain (GenBank No. KF772785.1) and were used to amplify the BVDV complete genomic sequence. The amplified products were cloned into a pMD-18T vector using pMD™ 18-T Vector Cloning Kit (TaKaRa, Beijing, China). Briefly, 4 μL of the purified amplification product was mixed with 1 μL of pMD18-T Vector and 5 μL of Solution I and incubated at 16 °C for 12 h. After the recombinant vector was transformed into DH5α competent cells (TaKaRa, Beijing, China), the single colony with AMP resistance was picked for amplification, and the plasmid was extracted using a TIANprep Mini Plasmid Kit (Tiangen, China) and submitted to Sangon Biotech Company (Shanghai, China) for DNA sequencing. To obtain the complete genomic sequence, the fragment sequences were trimmed and assembled using the SeqMan program in DNASTAR software.

### 2.5. Complete Genomic Sequence Analysis

He sequence alignment for the isolated BVDV, with the known BVDV strains listed in Table A2, was performed using Clustal W software. Phylogenetic analysis was carried out by the neighbor-joining method using MEGA7 software. The E2 glycosylation sites were analyzed using the NetNGlyc 1.0 Server (http://www.cbs.dtu.dk/services/NetNGlyc, accessed on 7 November 2022). The potential liner B cell epitopes on the E2 protein were predicted using the Bepipred Linear Epitope Prediction 2.0 method in IEDB Analysis Resource (http://tools.iedb.org/bcell/, accessed on 7 November 2022). Tertiary structures on the E2 protein of the new isolates were predicted using SWISS-MODEL (https://swissmodel.expasy.org/, accessed on 28 April 2023) and the images were generated using Pymol software.

### 2.6. Recombination Analysis

The recombination analysis between the new isolates and the reference strains were carried out using RDP4 software and SimPlot software. Seven methods, including RDP, GENECONV, Chimaera, MaxChi, Bootscan, SiScan, and 3Seq were used in RDP4. Fragments with recombination signals detected by at least four methods are considered to take place as recombination events.

## 3. Results

### 3.1. Virus Isolation and Identification

**Figure 1:**
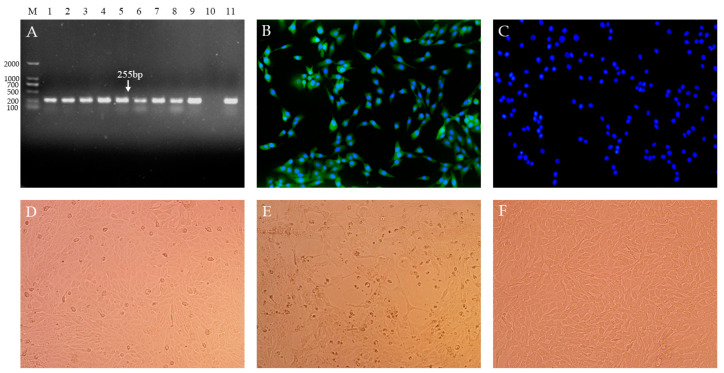
Nine BVDV isolates were isolated from serum samples of PI animals with mucosal disease. (**A**). Amplification signals with 255 bp were detected in all MDBK cells infected with the serum samples using RT-PCR. M. DNA Marker (D2000); 1–9. BVDV TJ2101–2109 infected cells; 10. Negative Control; 11. Positive Control. (**B**). The specific green signals were detected in infected cells with IFA. (**C**). Mock-infected cells were detected with IFA (400×). Cytopathic conditions of the NCP BVDV strain infected cells (**D**), CP BVDV strain infected cells (**E**), and mock-infected cells (**F**), were observed (200×).

Nine treated serum samples were cultured and passaged in MDBK cells. The presence of BVDV in the MDBK cells was confirmed with RT-PCR and an immunofluorescence assay. The amplification with 255 bp (Figure 1A) and the specific green signals (Figure 1B,C) were detected in all infected cells, suggesting the existence of BVDV. After five passages of the virus, nine isolates were obtained and named BVDV TJ2101, TJ2102, TJ2103, TJ2104, TJ2105, TJ2106, TJ2107, TJ2108, and TJ2109, respectively. Cytopathic effects were only observed in the TJ2102 and TJ2104 infected plates (Figure 1E), suggesting the BVDV TJ2102 and TJ2104 strains were CP BVDV strains, and the other isolates were NCP BVDV strains.

### 3.2. Genome Sequencing and Phylogenetic Analysis

**Figure 2:**
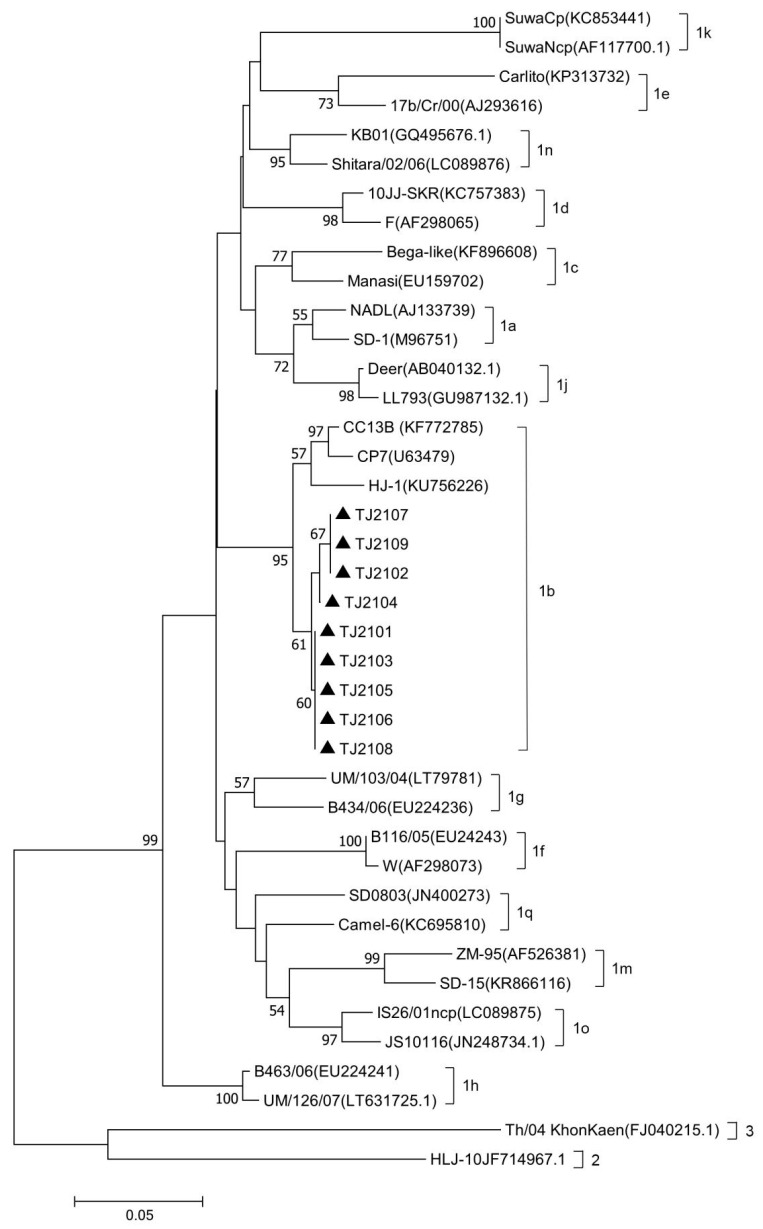
Phylogenetic analysis of the new isolates with 31 BVDV reference strains based on the 5′-UTR nucleotide sequences. The 5′-UTR nucleotide sequences of the new isolates were aligned with 31 reference BVDV strains, and phylogenetic analysis was carried out by the neighbor-joining method using MEGA7 software. The strains marked with black triangles were the new isolates. The 5′-UTR nucleotide sequences of the reference BVDV strains were obtained from the GenBank data library.

**Figure 3:**
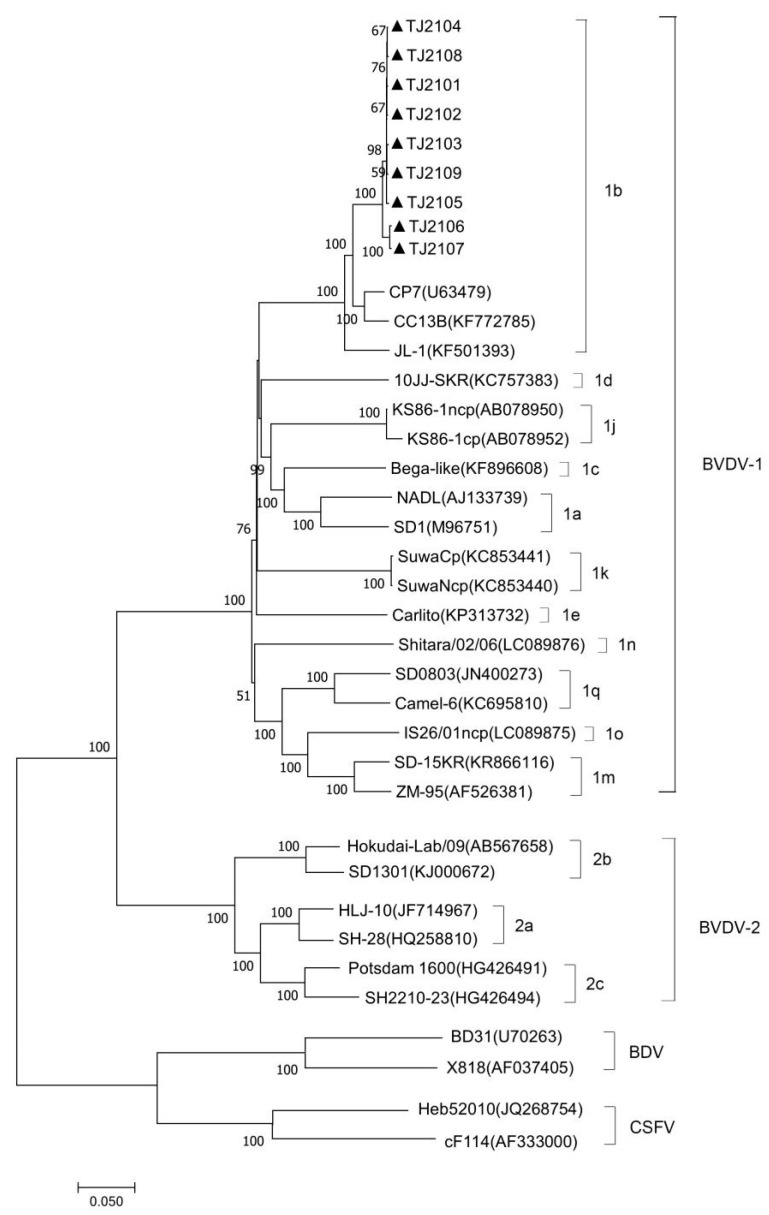
Phylogenetic analysis of the new isolates with pestiviruses based on the full-length nucleotide sequences. The genomic sequences of the new isolates were generated and aligned with representative pestiviruses including BVDV-1, BVDV-2, BDV, and CSFV, in this study. The strains marked with black triangles were the new isolates. The full-length genome nucleotide sequences of the reference pestivirus strains were obtained from the GenBank data library.

**Figure A1:**
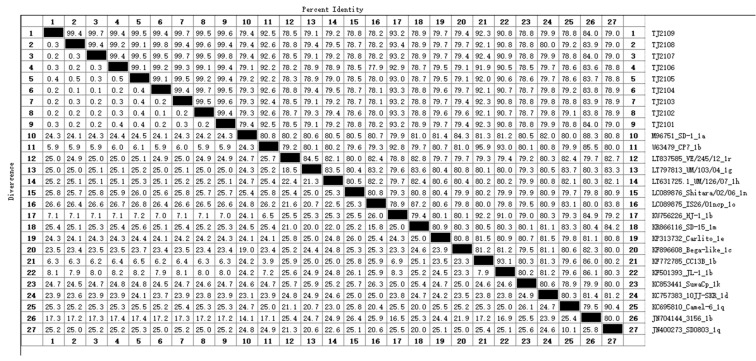
The genomic nucleotide identity between the new isolates and reference BVDV strains. The numbers from 1 to 27 represent the strains from TJ2109 to SD0803. The black cells represent the dividing line between percent identity and distance.

The complete genomic sequence of nine isolates were assembled and submitted to GenBank with accession numbers OR004805 to OR004813. The complete genomic sequence of the 9 new isolates were compared with 31 reference strains. Results showed that the genomic nucleotide identity between the new isolates and other BVDV strains was 78.5–92.6%, and the new isolates were most similar to the CP7 strain, sharing the 92.2–92.6% homology with it (Figure A1). As shown in Figure 2 and Figure 3, the new isolates and the CP7 and CC13B strains were in the same branch, suggesting that the new isolates could be classified as a BVDV-1b subgenotype.

### 3.3. Sequence Analysis of 5′-UTR

**Figure 4:**
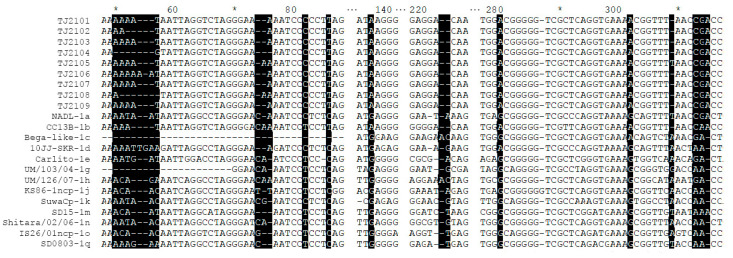
Sequence alignment of the 5′-UTR of the new isolates and 13 reference strains. Some nucleotide deletions or mutations of these isolates are shadowed and described in detail in the text. * represent a number. For example, the * between 60 and 80 is 70.

As shown in Figure 4, compared with the isolates of other subgenotypes, several molecular characteristics were found in the new isolates, including amino acid T at position 86 (T^86^) and A^137^, which were consistent with other 1b strains. In addition, several unique mutations, including nucleotide T82C in the 5′-UTR of the new isolates, BVDV-1d and BVDV-1k, and nucleotide G302A in the BVDV-1b and BVDV-1c strains, were found. Compared with the BVDV-1d strain (10JJ-SKR), TJ2102, TJ2104 and TJ2108 showed more continuous nucleotide deletions than other strains at positions 50–57. In addition, compared with the BVDV-1c and BVDV-1h strains, two nucleotide deletions were found in all of the BVDV-1b strains at positions 223–224, which had one more deletion than the strains of other subgenotypes. In addition, a nucleotide deletion at position 309 was observed in most BVDV-1b strains, which was not found in other strains.

### 3.4. Amino Acid Analysis of E2

**Figure 5:**
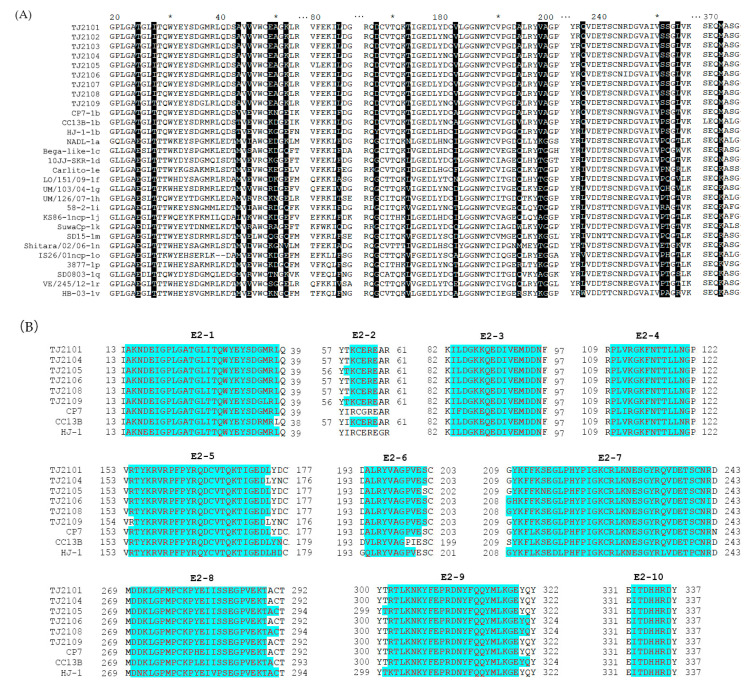

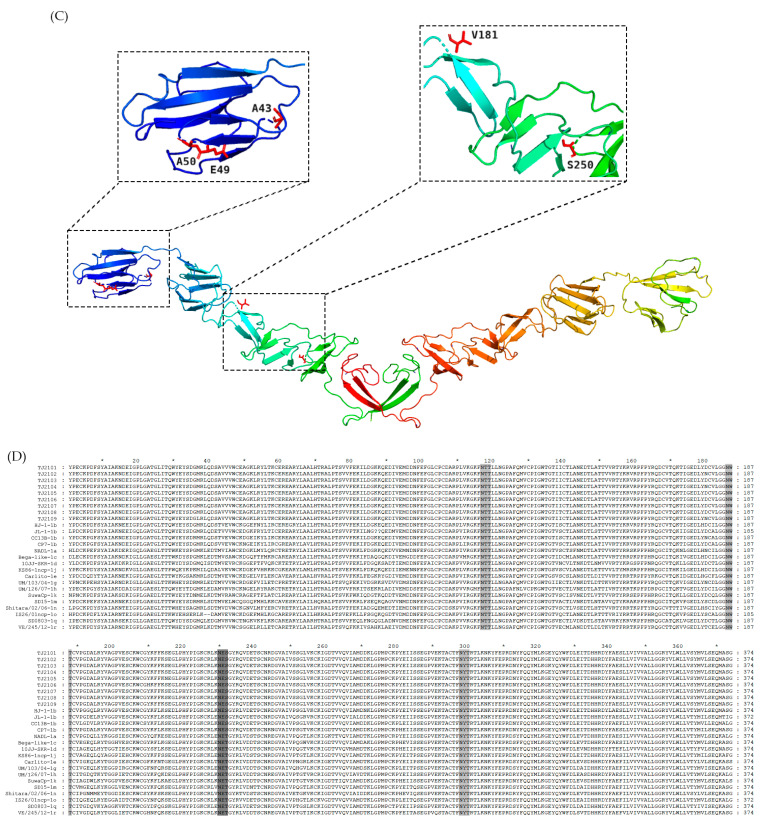
Amino acid characteristics of E2 protein. (**A**) Amino acid sequence alignment of E2 proteins of the nine new BVDV isolates and 20 reference isolates. Amino acid deletions or mutations of these isolates were shadowed and described in detail in the text. (**B**) The linear B cell epitopes on E2 proteins of the 9 new isolates and 3 BVDV-1b reference strains were predicted using BepiPred-2.0. The linear B cell epitopes on the E2 proteins of the TJ2102, TJ2103 and TJ2107 strains were not shown with the consistency of the TJ2101 strain. The amino acids of the predicted linear B cell epitopes are highlighted with red letters and blue shading. (**C**) Tertiary structure on the E2 protein of the new isolates. The unique amino acid residues are marked in red. (**D**) Glycosylation sites analysis of the new BVDV isolates and reference strains. The unique glycosylation site is shadowed darker than the conserved ones. * represent a number. For example, the * between 60 and 80 is 70.

As shown in Figure 5A, some molecular characteristics, including T^24^, T^173^, V^198^, A^199^, S^252^ and M^371^, were detected in the E2 protein of the new isolates, which was consistent with other BVDV-1b strains. In addition, I^27^, L^84^, D^167^, A^194^, Q^236^, and L^254^ were also found in the new isolates, which was similar to several BVDV-1b strains. Furthermore, the new isolates had several unique amino acid sequence characteristics, including A^43^, EA^49,50^, V^182^ and S^251^, which were different from other strains (Figure 5C). Compared with other BVDV-1b strains, CP7, CC13B and HJ-1, the amino acid replacements E45V and E52K were observed in the new strains. The potential linear B cell epitopes on the E2 protein of the new isolates and three BVDV-1b strains were predicted using BepiPred-2.0. As shown in Figure 5B, amino acid sequence variations affected 6 out of 10 liner B cell potential epitopes of the new isolate and the E2-2 epitope was found to be the most variant region, followed by E2-6, E2-8, and E2-9. The conserved regions were observed in the E2-1, E2-3, E2-4, and E2-10 epitopes. In addition, the glycosylation sites were conserved in the new isolates except 230 NET, which was consistent with other BVDV-1b strains (Figure 5D).

### 3.5. Amino Acid Analysis of E0

**Figure 6:**
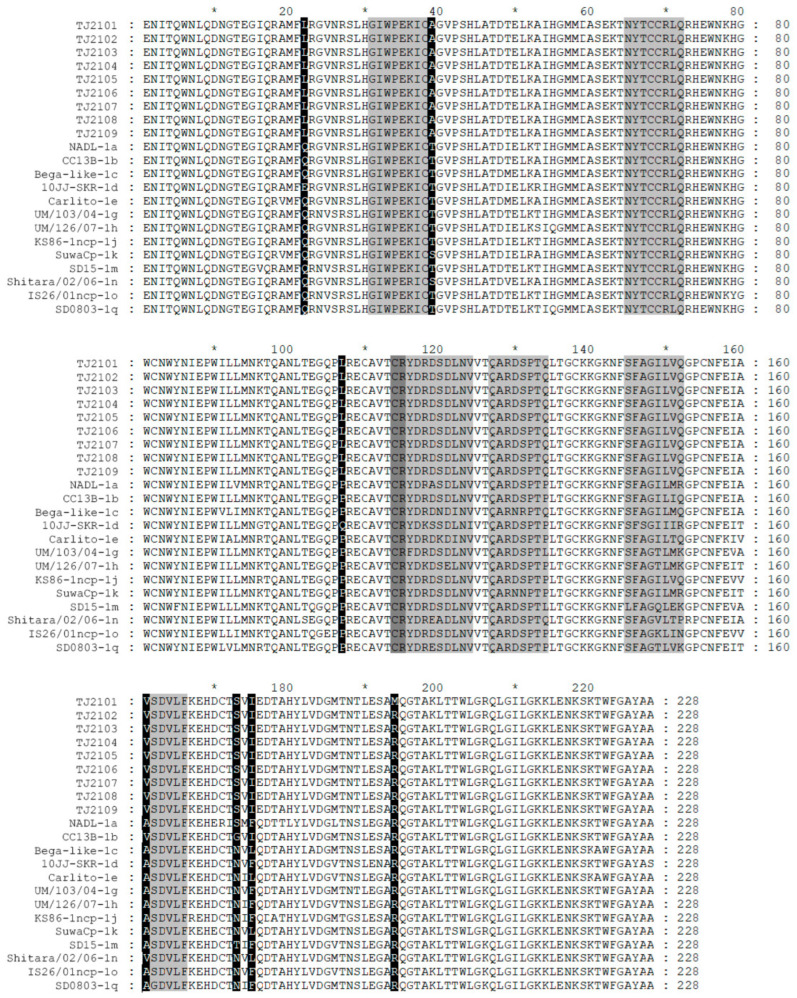
Amino acid sequence alignment of E0 of the 9 new BVDV isolates and 13 reference strains. Seven linear epitopes of E0 were found and are less lightly shadowed than the amino acid mutations region. * represent a number. For example, the * between 60 and 80 is 70.

E0 amino acid sequences of the new isolates were compared with strains of other BVDV subgenotypes. Seven linear epitopes exist in the BVDV E0 protein, including the epitopes 31GIWPEKIC38, 65NYTCCKLQ72, 127QARNRPTT134, 145SFAGTVIE152, 161VEDILY166, 114CRYDKNTDVNV124 and 116YDKNTDVNV124 [21]. As shown in Figure 6, all of the linear epitopes were found in the new isolates, while a unique amino acid replacement, a hydrophilic amino acid T mutating into a hydrophobic amino acid A, was only found at position 39 closed to the E0 epitope 31GIWPEKIC38 of the new isolates. The residues W^33^, L^71^, Q^127^, N^130^, S^145^, G^148^ and T^102^ were conserved in all BVDV-1 strains including the new isolates. However, an amino acid replacement at position 107 was only observed in the new isolates. In addition, several unique replacements, including the residues Q22L and P107L in the E0 of the new isolates, and R194M in the TJ2102 strain, were found. In addition, several amino acid sequence characteristics were observed, including S173 in the new isolates and BVDV-1a, I175 in BVDV-1b, and V161 in the BVDV-1b and BVDV-1o strains.

### 3.6. Recombination Analysis

**Figure 7:**
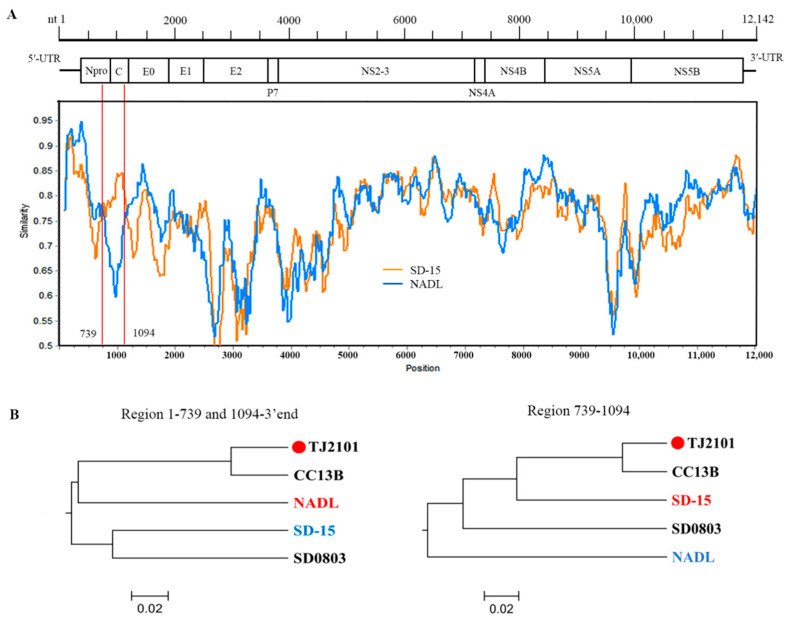
Recombination analysis on the genomes of the new BVDV isolates. Recombination analyses were conducted using RDP4 and SimPlot software. (**A**) Recombination breakpoints are marked with red lines, and the numbers beside the lines indicate the nucleotide position of the breakpoints relative to the genomic sequence of the recombinant virus. (**B**) Phylogenetic analyses of the recombination regions. Strains with red and blue letters were found to be major and minor parents of the recombinant strains.

A recombination analysis of the full-length genome sequences of the new isolates and reference strains was conducted using RDP4 and SimPlot software. As shown in Table 1, recombination signals were found in most new isolates except TJ2103 around the 691–1155 nucleotides region, suggesting most new isolates may be recombined by the major parent BVDV-1a NADL strain and the minor parent BVDV-1m SD-15 strain (Figure 7A,B).

## 4. Discussion

BVD is a significant bovine disease causing severe economic losses to the cattle industry. With the improvement of people’s living standards, the demand for beef and milk is increasing gradually. Multinational cattle transportation and commodity trading have also promoted the spread and mutation of the virus [5]. In recent years, the research on BVDV has been increasing, but most research is focused on the genotyping and epidemiological investigation of BVDV, while studies on the molecular characteristics and genome sequence of BVDV have been neglected. PI cattle will emit a large amount of virus during their lifetime, which seriously threatens the health of herds and is the main reason for the widely spread BVDV [34]. In this study, nine BVDV strains were isolated from the serum of PI cattle, and their full-length genome sequences and molecular characteristics were analyzed in order to provide a potential genome variation mode of the virus isolated from PI cattle.

The TJ2102 and TJ2104 strains were CP BVDV strains, and the other isolates were NCP BVDV strains. Surprisingly, no nucleotide insertions were found in the NS2-3 protein of the two CP BVDV isolates. Studies have shown that the insertion between NS2 and NS3 is necessary for its cleavage in CP BVDV strains [35], whether this characteristic of the TJ2102 and TJ2104 strains will affect the expression of NS3 remains to be further studied. In this study, the genome sequences of the nine isolates were compared with other reference strains. The new isolates had the highest similarity with the CP7 strain, sharing 92.2–96.3%, higher than other subgenotypes of BVDV such as BVDV-1o IS26/01ncp (77.9–78.2%) and BVDV-1n Shitara/02/06 (78.5–78.8%). The phylogenetic tree based on the genome sequence comparison showed that the new isolates were in the same branch with the BVDV-1b strains (CP7, JL-1 and CC13B), which was consistent with the analysis based on the 5′-UTR and N^pro^ sequences.

The 5′-UTR is a conserved region in pestiviruses that is often used to identify virus genotypes. In this study, some molecular characteristics (T^86^ and A^137^) were found in the 5′-UTR of every BVDV-1b strains. This finding could probably contribute to the identification of BVDV-1b strains. There exists an internal ribosomal entry site (IRES) in the secondary hairpin structure formed by the BVDV 5′-UTR, which is crucial for virus replication; changes in the secondary hairpin structure could directly affect the infectivity of the virus [36,37]. It has been found that deleting the 32–65 nucleotides or inserting 4 nucleotides at 63–66 in the stem-loop Ib did not affect the efficiency of the IRES, indicating that Ib was dispensable for BVDV translation. Deletions or insertions in any of the other four stem-loop regions, including II, IIIa, IIIc, and IIId, caused sharp decreases in IRES activity [38]. Therefore, deletions of the new isolates at the nucleotide positions 50–57 might not influence the IRES’s activity; although, whether the base mutations in other stem-loops of the new isolates affects BVDV translation remains to be confirmed experimentally.

Genome recombination plays an important role in viral genome diversification and genetic variation and affects pathogenicity and environmental adaptability. In this study, recombination events were identified using RDP4 and SimPlot software and were verified by at least four methods. Recombination signals were found in most of the new isolates except the TJ2103 strain. The major and minor parents of the recombinant strains were found to be BVDV-1a (the NADL strain isolated in USA, in 1988) and BVDV-1m (the SD-15 strain isolated from cattle in northern China, in 2015). As the center of the cattle industry in China, most of the cattle and dairy products were transported from Inner Mongolia to the eastern and central regions where the products were in high demand. Studies have shown that BVDV-1m might be the dominant subgenotype spreading in Inner Mongolia [39]. Therefore, the BVDV-1m strains might have been spread from Inner Mongolia to other regions. Moreover, in the past few decades, China has imported a large amount of Holstein semen and embryos from North America and Europe [40]. Therefore, we speculate that frequent commodity trade and transportation might directly or indirectly lead to the gene recombination and mutation of BVDV.

E0 is a relatively conserved structural protein in BVDV, containing seven linear epitopes, which can induce the production of neutralizing antibodies. Prediction based on hydrophilicity is a classic method used for predicting potential B cell epitopes; so changes in the hydrophilicity of amino acids may affect epitope predictions [41]. The amino acid at position 39 of the new isolates’ E0 protein had an opposite change in hydrophilicity; whether this change could affect the potential B cells’ epitopes remains to be further verified. For CSFV, the residues W^33^, L^71^, Q^127^, N^130^, S^145^, G^148^, T^102^ and D^107^ may be crucial for interactions with antibodies [42] In the present study, one amino acid replacement, P107L, has been found in the E0 protein of the new isolates, which could influence the effectiveness of vaccination. In addition, binding to the cell surface glycosaminoglycans (GAGs) and attachment to the cell surface of E0 plays an important role in the viral infection of host cells [22,23]. The cluster of residues 480KKLENKSK487 localized at the C-terminal end of E0 was proved to be a binding site of GAGs. Replacements at the amino acids 481 and 485 in E0 resulted in the loss of the ability to bind to cells and even the failure to infect cells [24]. The new isolates were found to be conserved in this region.

The E2 protein is an envelope glycoprotein in BVDV that is necessary for virus recognition, adsorption, and the entry into host cells. After the C-terminus of E0 binds to cell surface glycosaminoglycans (GAGs), E2 binds to CD46 to allow the virus to enter the cell [43,44]. E2 is the most antigenic protein in BVDV, and it is the main site that induces the production of neutralizing antibodies [45]. In the present study, some unique molecular features were found in the new isolates. Whether the epitopes are arranged in the correct conformation determines the efficiency of antibody binding. A change of a certain amino acid residue may affect the sequence or three-dimensional structure of the epitope [46]. These amino acid variations altered eight B cell linear epitopes of the isolate’s E2 protein, especially around position 60. Linear epitopes of viral proteins affect the antigenic structure and virus–antibody interactions, and the changes may affect the effectiveness of vaccination. Some special epitopes can be used in the development of new vaccines but the antigenic epitopes predicted using software cannot fully reflect the actual site of virus-induced neutralizing antibody production [47]. N-glycosylation is the most common form of protein glycosylation and has two forms, Asn-X-Ser and Asn-X-Thr. In the present study, a NET-to-NES substitution at position 230 was found in E2 of the new isolates and in other BVDV-1b strains. Studies have found that the replacement of serine with threonine greatly reduces the efficiency of N-glycosylation [48]. Whether this change affects the structure and function of the E2 protein remains to be further verified.

Consistent with previous findings, the BVDV genome isolated from PI cattle had more substitutions, and these amino acid substitutions mainly occurred in the structural proteins E0 and E2 [49]. In the present study, some molecular characteristics of PI bovine isolates were found; whether these features are conserved in PI bovine isolates of other subgenotypes needs further study.

## 5. Conclusions

In this study, nine isolates were isolated from PI calves and identified as the BVDV-1b subgenotype. Some unique variations that may be related to viral replication and immune response were found in the new isolates. A novel B cell potential linear epitope was found in the E2 protein of the new isolates, suggesting that the new isolate may be a potential candidate for vaccine optimization. Recombination analysis suggested that commodity trade across regions may have contributed to the emergence of viral diversity. Whether these unique molecular characteristics are conserved in the PI isolates of other subgenotypes remains to be further verified. This study provides a potential genetic variation model of PI isolates in order to provide a reference for the genetic evolution of BVDV.

## Figures and Tables

**Table 1:** Analysis of recombination events in the genome of the new isolates using RDP4 software.

Recombinant Strains	Location(s) [nt(99%CI)]	Major Parent	Minor Parent				Methods ^a^			
				RDP	GeneConv	Bootscan	MaxChi	Chimaera	SiScan	3Seq
TJ2101	739–1094	NADL (1a)	SD-15 (1m)	1.140 × 10^−1^	-	3.668 × 10^−3^	9.396 × 10^−3^	1.006 × 10^−2^	1.909 × 10^−4^	-
TJ2102	739–1211	NADL (1a)	SD-15 (1m)	1.369 × 10^−4^	-	5.171 × 10^−5^	1.518 × 10^−4^	3.415 × 10^−5^	1.969 × 10^−3^	-
TJ2104	691–1155	NADL (1a)	SD-15 (1m)	5.845 × 10^−5^	-	3.728 × 10^−5^	7.622 × 10^−5^	2.269 × 10^−5^	1.857 × 10^−3^	-
TJ2105	692–1164	NADL (1a)	SD-15 (1m)	1.411 × 10^−3^	-	1.322 × 10^−3^	1.490 × 10^−3^	2.352 × 10^−4^	-	-
TJ2106	724–1188	NADL (1a)	SD-15 (1m)	7.965 × 10^−5^	-	5.366 × 10^−5^	1.409 × 10^−4^	3.420 × 10^−5^	3.236 × 10^−3^	-
TJ2107	691–1156	NADL (1a)	SD-15 (1m)	1.469 × 10^−4^	-	1.701 × 10^−4^	1.520 × 10^−4^	5.010 × 10^−5^	3.015 × 10^−3^	-
TJ2108	691–1155	NADL (1a)	SD-15 (1m)	5.361 × 10^−5^	-	3.505 × 10^−5^	3.468 × 10^−5^	1.485 × 10^−5^	1.559 × 10^−3^	-
TJ2109	691–1155	NADL (1a)	SD-15 (1m)	1.074 × 10^−4^	-	4.368 × 10^−5^	1.012 × 10^−4^	3.418 × 10^−5^	2.724 × 10^−2^	-

^a^ Seven methods implemented in the RDP4 software were used to analyze the potential recombination events. The numbers under the methods represent the average *p*-values obtained using the methods to identify potential recombination events.

## Data Availability

The complete genome sequences of the nine BVDV isolates (Accession number: OR004805-OR004813) have been submitted to GenBank.

## Appendix A

**Table A1:** Primers used for the amplification of the complete genome sequence.

Primers		Primer Sequence (5′-3′)	Annealing Temperature
P1	F	GTATACGAGGTTAGGCAAGTTCTCG	52 °C
	R	GCAGCATCCTATCAGACTGTATTC	
P2	F	AAAAGAGGCTAACCACG	47 °C
	R	CCATGT(C)TTG(A)TTCCACTCAT	
P3	F	CCTCAAGAGTCACGCAAGAAA	52 °C
	R	TACGGTCCCTGTCCATCCTAT	
P4	F	TCTTTGCCCATGCGATGCTAG	55 °C
	R	GCTCCGAATCAGGAACACCC	
P5	F	CGCTACTGATGATTAGTTATGTG	50 °C
	R	TATGTTTACTGCCTCTGGATT	
P6	F	GCCAAAGCAACAACAAGTTAA	52 °C
	R	AGGGACCTGGACCTCATAACT	
P7	F	TGAAGTAGCCAAGAAACTAAAAGC	54 °C
	R	GTCTGACACCGACTCACCCC	
P8	F	TTTATGCCACCGAAGATG	48 °C
	R	TGACAATACCGTGCTCCA	
P9	F	GGACTCTGAAGGGAAGATAAG	52 °C
	R	TGATGCCTGAAGAAACCAACT	
P10	F	CGGACCAAACAAGAATAGTGA	52 °C
	R	GCTGGTATTATCAGACCACCTAA	
P11	F	AGGGGCTGCAGGCTTTCTA	55 °C
	R	TTTTTCCCCTTCGGTTATCTTT	
P12	F	CGGGCAAGCCTCAAAAGATAA	58 °C
	R	CTCGCCTGCAGCTGAAGTTGT	

**Table A2:** Reference pestiviruses used for the sequence and phylogenetic analyses.

Pestivirus Species	Subtype	Strain	Origin	Location	Collection Year	GenBank Accession Number
BVDV-1	1a	NADL	Cattle	USA	1963	AJ133739
BVDV-1	1a	SD1		USA		M96751
BVDV-1	1b	CC13B	Cattle	China	2013	KF772785
BVDV-1	1b	CP7	Cattle	Germany	1987	U63479
BVDV-1	1b	JL-1	Cattle	China	2009	KF501393
BVDV-1	1c	Bega-like	Bovine	Australia	2012	KF896608
BVDV-1	1d	10JJ-SKR	Cattle	South Korea	2010	KC757383
BVDV-1	1e	Carlito	Cattle	Switzerland	2014	KP313732
BVDV-1	1h	UM/126/07	Cattle	Italy	2016	LT631725
BVDV-1	1j	KS86-1ncp	Cattle	Japan	2002	AB078950
BVDV-1	1j	KS86-1cp	Cattle	Japan	2002	AB078952
BVDV-1	1k	SuwaCp	Cattle	Switzerland	1999	KC853441
BVDV-1	1k	SuwaNcp	Cattle	Switzerland	2000	KC853440
BVDV-1	1m	SD-15	Cattle	China	2015	KR866116
BVDV-1	1m	ZM-95	Pig	China	1995	AF526381
BVDV-1	1n	Shitara/02/06	Cattle	Japan	2006	LC089876
BVDV-1	1o	IS26/01ncp	Cattle	Japan	2001	LC089875
BVDV-1	1q	Camel-6	Camel	China	2010	KC695810
BVDV-1	1q	SD0803	Pig	China	2008	JN400273
BVDV-1	1r	VE/245/12	-	Italy	2012	LT837585
BVDV-2	2a	HLJ-10	Cattle	China	2011	JF714967
BVDV-2	2a	SH-28	Pig	China	2009	HQ258810
BVDV-2	2b	Hokudai-Lab/09	Bovine	Japan	2010	AB567658
BVDV-2	2b	SD1301	Cattle	China	2012	KJ000672
BVDV-2	2c	Potsdam 1600	Cattle	Germany	2000	HG426491
BVDV-2	2c	SH2210-23	Cattle	Germany	2010	HG426494
BDV	BDV	BD31	Lamb	USA	-	U70263
BDV	BDV	X818	Sheep	Germany	-	AF037405
CSFV	CSFV	Heb52010	-	China	2012	JQ268754
CSFV	CSFV	cF114	-	China	2001	AF333000
